# A comparison of computationally predicted functional metagenomes and microarray analysis for microbial P cycle genes in a unique basalt-soil forest

**DOI:** 10.12688/f1000research.13841.1

**Published:** 2018-02-12

**Authors:** Erick S. LeBrun, Sanghoon Kang

**Affiliations:** 1 Center for Reservoir and Aquatic Systems Research, Department of Biology, Baylor University, Waco, TX, 76798-7388, USA

**Keywords:** Metagenome, phosphorus, microbial communities, MiSeq, PICRUSt, GeoChip, nutrient cycling

## Abstract

Here we compared microbial results for the same Phosphorus (P) biogeochemical cycle genes from a GeoChip microarray and PICRUSt functional predictions from 16S rRNA data for 20 samples in the four spatially separated Gotjawal forests on Jeju Island in South Korea. The high homogeneity of microbial communities detected at each site allows sites to act as environmental replicates for comparing the two different functional analysis methods. We found that while both methods capture the homogeneity of the system, both differed greatly in the total abundance of genes detected, as well as the diversity of taxa detected. Additionally, we introduce a more comprehensive functional assay that again captures the homogeneity of the system but also captures more extensive community gene and taxonomic information and depth. While both methods have their advantages and limitations, PICRUSt appears better suited to asking questions specifically related to microbial community P as we did here. This comparison of methods makes important distinctions between both the results and the capabilities of each method and can help select the best tool for answering different scientific questions.

## Introduction

Relating the functionality of microbes to environmental factors is one of the primary goals in microbial ecology. With the advent of modern genomic technologies, such as next generation sequencing and microarray hybridization, there are more options than ever to test environmental community’s genomics and functional capabilities. Metagenome sequencing is one of the most thorough and comprehensive methods currently available for looking at microbial community gene compositions ^1– 5^, but can be costly and generate enormous data sets that require a large amount of work in processing, analysis, and storage. Two technologies currently in use for looking at community functional profiles that can be less expensive and more accessible than metagenome sequencing include computationally predicted functional metagenomes (PFMs) ^6^ and microarray analyses ^7^. These technologies both have known advantages and disadvantages ^8^, but investigation into how they compare in the same system is still needed.

Here we compare PFMs from Phylogenetic Investigation of Communities by Reconstruction of Unobserved States (PICRUSt) ^6^ to GeoChip ^9^ microarray data. While both methods are distinct, they can each be applied to an environmental community gene pool to estimate the presence and abundance of genes within the community genomic landscape related to function. Resulting datasets from each technique are tables showing counts of genes or functions as determined by either probes (microarray) or reference data (PFMs), and therefore are directly comparable in the context of functional gene landscapes within the system. We utilize 20 sites in a unique basalt-soil Gotjawal forest on Jeju Island in Korea. Despite being both rocky, lava-formed basalt and having dense vegetation ^10^, this forest is considered a wetland environment due to the homogenous, rocky soil and its capacity for absorbing water ^11^. All 20 sites, though spatially separated by distance of 5 km to 65 km ( Figure S1), showed strong homogeneity in bacterial/archaeal community assemblies in 16S rRNA gene taxonomic analysis ( Figure S2) and so act as replicates in this system for the current study. This makes it ideal for comparing the technologies. We specifically look at how these technologies perform related to the same phosphorus (P) cycle genes as the unique basalt-soil environment has the potential to be a unique P environment ^12– 14^.

## Methods

### Data origination and processing

GeoChip 4.0 data for P cycle genes came from Kim *et al.* ^15^. For sequencing data, we started with raw sequencing files also from the study by Kim *et al.* ^16^. Paired-end reads were combined using the join-fastq algorithm from eautils ^17^. Un-paired reads were discarded at this time. Additional sequence processing was performed using Quantitative Insights Into Microbial Ecology (QIIME) version 1.9.1 ^18^. Sequences were then filtered with a maximum unacceptable Phred quality score of 20. Chimeric sequences were identified and removed using the UCHIME algorithm within USEARCH ^19^. Operational taxonomic unit (OTU) picking was performed via open reference using uclust against the Greengenes 13_8 database with a 0.97 similarity cutoff ^20^. Singleton sequences were removed during OTU picking and taxonomy was assigned with Greengenes 13_8 database as reference.

Only reads identified in closed reference picking were used for the PICRUSt analysis. Using PICRUSt ^6^, predicted functional metagenomes (PFMs) were constructed from the resulting 16S rRNA sequences. PFMs were generated using the Kyoto Encyclopedia of Genes and Genomes (KEGG) database ^21, 22^ as a functional reference.

### Genes studied

The GeoChip 4.0 data provided probe data for genes identified as “phytase”, “ppk”, and “ppx”. We identified these genes in the KEGG database to have the KEGG orthology (KO) numbers K01083 and K01093 for phytase, K00937 for ppk, and K01514 for ppx These KO numbers were the only PICRUSt results extracted for direct comparison. Additionally, we built another P assay in PICRUSt utilizing 417 KO numbers associated with P ( Table S1).

### Statistical analyses

All analyses were performed in the R software package v.3.2.3 ^23^. The relationship between the PICRUSt and GeoChip data was tested using a Mantel test with the Pearson correlation method and 1,000 permutations through the vegan package ^24^. Non-metric multidimensional scaling (NMDS) ordinations were constructed using Bray-Curtis dissimilarity through the vegan package. A PROcrustean randomization TEST of community environment concordance (PROTEST), a potentially more sensitive detection method than a Mantel test, was also used to compare the NMDS ordinations to each other ^25^. Figures and plots were created using the ggplot2 package ^26^.

## Results and discussion

**Figure 1.**
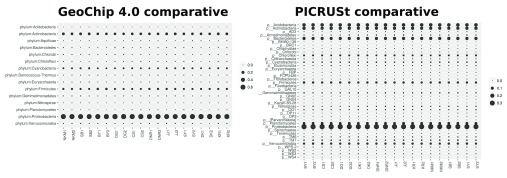
Bubble plots of taxa relative abundance detected by the GeoChip 4.0 array PICRUSt from 16S rRNA data for P cycle genes found on GeoChip array.

**Figure 2.**
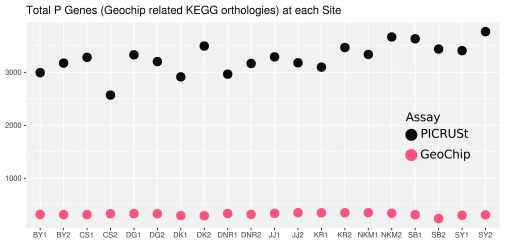
Plot of total P cycle gene counts as detected by PICRUSt and GeoChip at each site.

Both PICRUSt and GeoChip appear to have captured the homogeneity of the system ( Figure 1). PICRUSt captured much more diversity and depth in terms of taxa identified ( Figure 1) and total counts ( Figure 2) than GeoChip. PICRUSt identified organisms from 40 different phyla where GeoChip identified organisms from 15. Total counts at each site for the two methods were on a very different scale. When placed on a scale that shows the variation in each set of counts, it becomes apparent that the trends of total counts across sites do not match between methods ( Figure S3). The Mantel test resulted in no significant statistic between the two data sets and Procrustes analysis confirmed this, showing no significant correlation either ( Figure S4). The same analyses were performed with the data for each gene isolated and each of the three genes independently provided similar results of inconsistency between methods to the comparison of total gene datasets. There was no correlation between the datasets in Mantel or Procrustes analysis and gene counts and trends were markedly different.

The new PICRUSt assay with 417 P related genes captured the system homogeneity but with additional depth ( Figure S5). The new assay identified organisms from 41 phyla similar to the smaller, comparative assay’s 40 but also provided data counts per site ranging from ~70,000 to ~110,000. The PICRUSt dataset from the new assay not only represents what is likely a better dataset for answering community functional questions within the P cycle than the previous, comparative PICRUSt or GeoChip datasets, but also illustrates an important difference between the two methods. While both methods could be considered “closed-format” technologies in that they are reliant on the available known references ^8^, the process of adapting or updating the two methods contrasts. The method of using computational predictions is highly adaptable and allows for the easy inclusion or exclusion of additional genes ^6^. Improving or expanding the reference database that computational prediction can be achieved through simply updating the curated reference database. The microarray method is more involved including the identification, creation, and inclusion of specific target probes into the manufacturing of a microarray ^7^.

It is important to note that for our comparison we are specifically looking at functional genes within the P biogeochemical cycle. Both methods explored are designed for, and capable of looking a more comprehensive whole functional profile for communities. Computational functional prediction seems to be better suited to the task of viewing independent functional groupings as we did here. While microarrays have shown linear relationships to RNA and DNA levels in environmental systems ^16, 27^, they are limited in coverage and small sequence divergence can affect quantitative capability ^7^. These quantitative limitations should be carefully considered in light of recent findings showing that the composition of P cycle genes in some microbial communities are more closely related to environmental P levels than absolute abundance ^1^. Computational functional prediction again seems better equipped to handle questions related to functional gene composition due to the high specificity of probes to taxa and limited genes included in microarrays. It is also important to note that the data from both methods is representative of DNA present in microbial communities and not true expression levels or enzyme abundance.

## Conclusions

Computational functional prediction and microarray analysis of P cycle genes both captured system homogeneity. However, they did not agree in terms of capturing absolute abundance or taxonomic composition in P cycle genes. Computational functional prediction provided more count depth and taxonomic diversity than microarray analysis did. The ease with which computational functional prediction is adapted additionally allowed for the capture of additional genes and taxonomic diversity in P function along with increased depth by expanding the PICRUSt assay to include 417 KO numbers related to P function instead of the original 4 used in the microarray comparison. While we compared two methods for the exploration of functional P cycle genes within microbial communities to each other, an additional comparison to whole metagenome data in a system would further validate either method.

## Data availability

The sequence data used in this study was deposited in the NCBI Sequence Read Archive (SRA) under the BioSample accession numbers SAMN06049757 to SAMN06049776. The GeoChip microarray data used in this study is available in OSF: http://doi.org/10.17605/OSF.IO/AT93H ^28^.

Data are available under the terms of the Creative Commons Attribution 4.0 International license (CC-BY 4.0).

## Supplemental material

Supplementary File 1: File containing all supplementary figures referenced in main text ( Figure S1–S5).

Supplementary File 2: File containing the supplementary table referenced in main text ( Table S1).
