# Supplementary material for: A comparison of computationally predicted functional metagenomes and microarray analysis for microbial P cycle genes in a unique basalt-soil forest
Source: F1000Res. 2018 Feb 12;7:179. [Version 1] doi: 10.12688/f1000research.13841.1 (PMC6051228; doi:10.12688/f1000research.13841.1)

Supplementary Information: Supplementary Figures

A comparison of computationally predicted functional metagenomes and microarray analysis for microbial P cycle genes in a unique basalt-soil forest.

Erick S. LeBrun[1]* and Sanghoon Kang[1]

[1] Center for Reservoir and Aquatic Systems Research, Department of Biology, Baylor University, Waco, Texas 76798-7388 USA

*Corresponding author:

Erick S. LeBrun

Department of Biology

Baylor University

One Bear Place 97388

Waco, Texas 76798-7388 USA

Erick_LeBrun@Baylor.edu

Conflict of Interest Statement:

The authors declare no conflict of interest.

Supplementary Figures

Figure S1 Map of Gotjawal forest sample sites on Jeju Island, Korea. Originally published in Kim, J.-S. et al. Microbial Community Structure and Functional Potential of Lava-Formed Gotjawal Soils in Jeju, Korea. (In review)

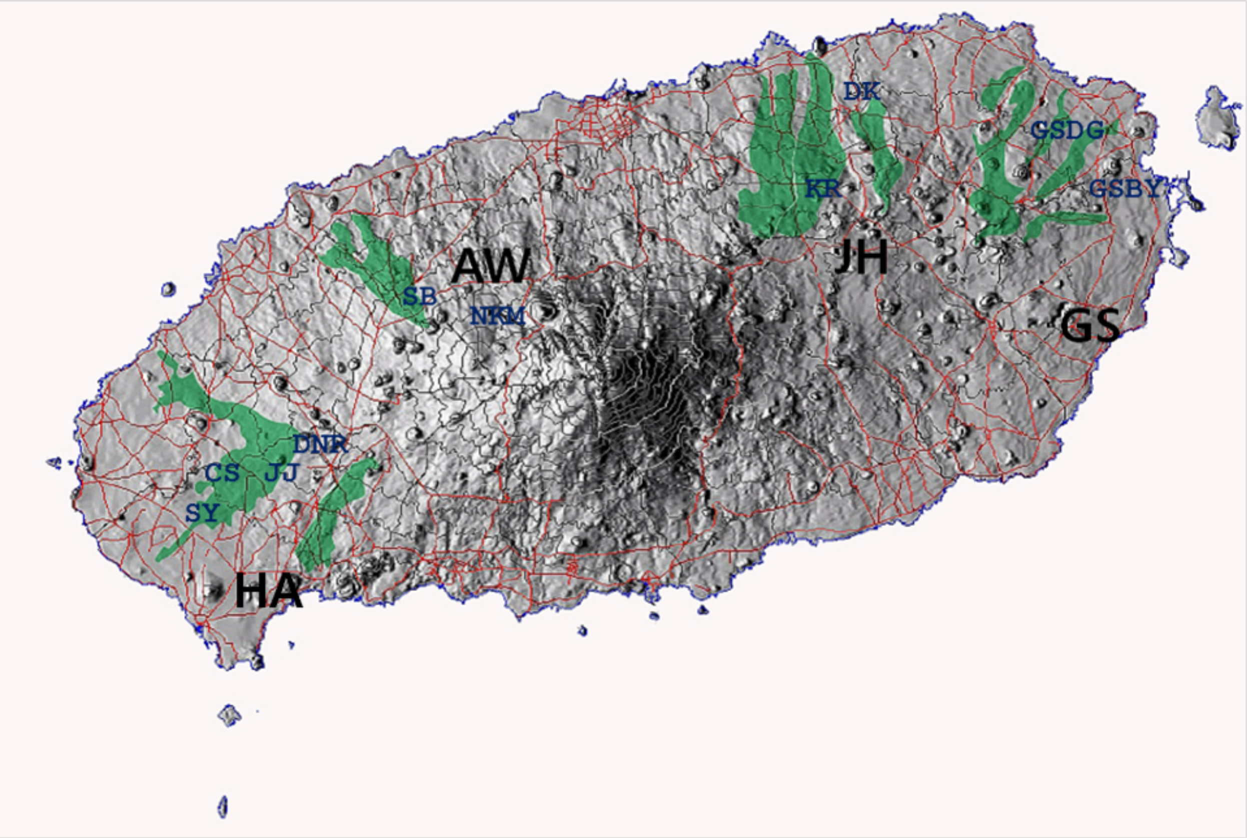

Figure S2 Bar plot of taxa at the phyla level in the system showing taxonomic homogeneity across sites. Rarefaction depth of 41,000 used.

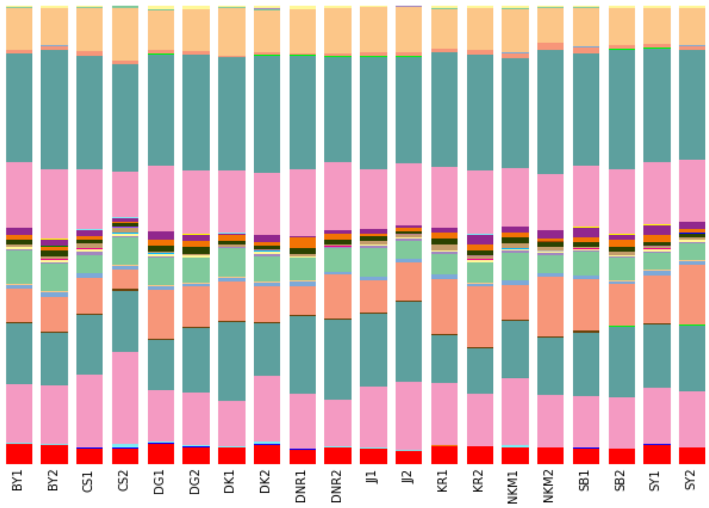

Legend	Taxonomy	%	%	%	%	%	%	%	%	%	%	%	%	%	%	%	%	%	%	%	%	%
	Unassigned;Other	3.7%	4.4%	4.1%	3.5%	3.3%	4.6%	3.8%	3.7%	4.2%	3.3%	3.7%	3.5%	2.9%	4.0%	3.9%	3.6%	3.6%	3.5%	3.4%	4.3%	3.7%
	k__Archaea;p__Crenarchaeota	0.0%	0.0%	0.1%	0.1%	0.3%	0.0%	0.0%	0.0%	0.1%	0.0%	0.0%	0.0%	0.0%	0.0%	0.0%	0.0%	0.0%	0.0%	0.0%	0.0%	0.0%
	k__Archaea;p__Euryarchaeota	0.0%	0.0%	0.0%	0.0%	0.0%	0.0%	0.0%	0.0%	0.1%	0.0%	0.0%	0.0%	0.0%	0.0%	0.0%	0.0%	0.0%	0.0%	0.0%	0.0%	0.0%
	k__Archaea;p__[Parvarchaeota]	0.0%	0.0%	0.0%	0.0%	0.0%	0.0%	0.0%	0.0%	0.0%	0.0%	0.0%	0.0%	0.0%	0.0%	0.0%	0.0%	0.0%	0.0%	0.0%	0.0%	0.0%
	k__Bacteria;Other	0.0%	0.0%	0.0%	0.0%	0.0%	0.0%	0.0%	0.0%	0.0%	0.0%	0.0%	0.0%	0.0%	0.0%	0.0%	0.0%	0.0%	0.0%	0.0%	0.0%	0.0%
	k__Bacteria;p__	0.0%	0.0%	0.0%	0.0%	0.0%	0.0%	0.0%	0.0%	0.0%	0.0%	0.0%	0.0%	0.0%	0.0%	0.0%	0.0%	0.0%	0.0%	0.0%	0.0%	0.0%
	k__Bacteria;p__AD3	0.2%	0.3%	0.2%	0.1%	0.8%	0.3%	0.2%	0.1%	0.5%	0.1%	0.0%	0.1%	0.1%	0.2%	0.0%	0.4%	0.0%	0.1%	0.0%	0.0%	0.0%
	k__Bacteria;p__Acidobacteria	12.8%	12.8%	12.7%	15.9%	20.2%	11.2%	11.5%	9.9%	14.4%	11.9%	10.1%	13.4%	14.9%	13.6%	11.4%	14.6%	11.5%	11.1%	11.3%	12.3%	12.1%
	k__Bacteria;p__Actinobacteria	13.8%	13.2%	11.4%	13.0%	13.3%	10.9%	14.2%	17.3%	11.5%	17.1%	17.6%	15.8%	17.6%	10.4%	9.8%	12.6%	12.4%	13.9%	15.2%	13.9%	14.4%
	k__Bacteria;p__Armatimonadetes	0.2%	0.3%	0.3%	0.2%	0.3%	0.2%	0.1%	0.1%	0.2%	0.1%	0.1%	0.2%	0.2%	0.3%	0.3%	0.3%	0.3%	0.3%	0.2%	0.1%	0.1%
	k__Bacteria;p__BHI80-139	0.0%	0.0%	0.0%	0.0%	0.0%	0.0%	0.0%	0.0%	0.0%	0.0%	0.0%	0.0%	0.0%	0.0%	0.0%	0.0%	0.0%	0.0%	0.0%	0.0%	0.0%
	k__Bacteria;p__BRC1	0.0%	0.0%	0.0%	0.0%	0.0%	0.0%	0.0%	0.0%	0.0%	0.0%	0.0%	0.0%	0.0%	0.0%	0.1%	0.0%	0.0%	0.0%	0.0%	0.0%	0.0%
	k__Bacteria;p__Bacteroidetes	9.2%	7.1%	7.5%	7.8%	4.2%	10.7%	8.8%	8.7%	7.7%	6.4%	9.6%	7.1%	8.3%	12.0%	13.1%	7.4%	13.1%	11.3%	9.2%	10.4%	13.3%
	k__Bacteria;p__Chlamydiae	0.8%	1.0%	1.1%	1.0%	0.9%	0.8%	0.7%	0.9%	0.8%	1.1%	0.6%	0.8%	0.8%	0.9%	0.7%	0.9%	0.6%	0.6%	0.6%	0.9%	0.7%
	k__Bacteria;p__Chlorobi	0.2%	0.2%	0.2%	0.2%	0.2%	0.2%	0.2%	0.2%	0.3%	0.3%	0.2%	0.1%	0.1%	0.1%	0.2%	0.2%	0.1%	0.2%	0.1%	0.3%	0.2%
	k__Bacteria;p__Chloroflexi	5.1%	7.2%	6.1%	3.9%	6.2%	6.0%	5.4%	6.2%	5.4%	4.7%	5.1%	6.2%	3.9%	4.5%	4.3%	6.0%	3.9%	5.1%	5.0%	3.8%	3.9%
	k__Bacteria;p__Cyanobacteria	0.3%	0.4%	0.3%	0.6%	0.3%	0.2%	0.2%	0.2%	0.4%	0.2%	0.2%	0.5%	0.3%	0.3%	0.2%	0.4%	0.4%	0.3%	0.2%	0.3%	0.2%
	k__Bacteria;p__Elusimicrobia	0.4%	0.3%	0.5%	0.7%	0.5%	0.5%	0.4%	0.1%	0.5%	0.2%	0.2%	0.4%	0.3%	0.3%	0.5%	0.3%	0.5%	0.5%	0.3%	0.5%	0.4%
	k__Bacteria;p__FBP	0.0%	0.0%	0.0%	0.0%	0.0%	0.0%	0.0%	0.0%	0.0%	0.0%	0.0%	0.0%	0.0%	0.0%	0.0%	0.0%	0.0%	0.0%	0.0%	0.0%	0.0%
	k__Bacteria;p__FCPU426	0.1%	0.1%	0.1%	0.1%	0.1%	0.2%	0.1%	0.0%	0.2%	0.0%	0.0%	0.1%	0.0%	0.1%	0.1%	0.0%	0.1%	0.1%	0.0%	0.1%	0.1%
	k__Bacteria;p__Fibrobacteres	0.0%	0.0%	0.0%	0.0%	0.0%	0.0%	0.1%	0.0%	0.1%	0.0%	0.0%	0.0%	0.0%	0.1%	0.1%	0.0%	0.1%	0.0%	0.1%	0.0%	0.1%
	k__Bacteria;p__Firmicutes	0.6%	0.5%	0.5%	1.1%	1.3%	0.3%	0.2%	0.5%	0.2%	0.5%	0.3%	0.7%	0.7%	1.2%	0.5%	1.0%	0.7%	0.3%	0.6%	0.5%	0.4%
	k__Bacteria;p__Fusobacteria	0.0%	0.0%	0.0%	0.0%	0.0%	0.0%	0.0%	0.0%	0.0%	0.0%	0.0%	0.0%	0.0%	0.0%	0.0%	0.0%	0.0%	0.0%	0.0%	0.0%	0.0%
	k__Bacteria;p__GAL15	0.0%	0.0%	0.0%	0.0%	0.0%	0.0%	0.0%	0.0%	0.0%	0.0%	0.0%	0.0%	0.0%	0.0%	0.0%	0.0%	0.0%	0.0%	0.0%	0.0%	0.0%
	k__Bacteria;p__GN02	0.0%	0.1%	0.0%	0.0%	0.0%	0.0%	0.0%	0.0%	0.0%	0.0%	0.0%	0.0%	0.0%	0.0%	0.1%	0.0%	0.0%	0.0%	0.0%	0.0%	0.0%
	k__Bacteria;p__GN04	0.0%	0.0%	0.0%	0.0%	0.0%	0.0%	0.0%	0.0%	0.1%	0.0%	0.0%	0.0%	0.0%	0.0%	0.0%	0.0%	0.0%	0.0%	0.0%	0.0%	0.0%
	k__Bacteria;p__GOUTA4	0.0%	0.0%	0.0%	0.0%	0.0%	0.0%	0.0%	0.0%	0.0%	0.0%	0.0%	0.0%	0.0%	0.0%	0.0%	0.0%	0.0%	0.0%	0.0%	0.0%	0.0%
	k__Bacteria;p__Gemmatimonadetes	1.0%	0.9%	1.2%	0.9%	0.9%	1.3%	1.4%	0.7%	0.8%	1.2%	1.0%	0.9%	0.7%	1.3%	1.1%	1.3%	1.1%	1.0%	1.2%	0.9%	0.9%
	k__Bacteria;p__Kazan-3B-28	0.0%	0.0%	0.0%	0.0%	0.0%	0.0%	0.0%	0.0%	0.0%	0.0%	0.0%	0.0%	0.0%	0.0%	0.0%	0.0%	0.0%	0.0%	0.0%	0.0%	0.0%
	k__Bacteria;p__MVP-21	0.0%	0.0%	0.0%	0.0%	0.0%	0.0%	0.0%	0.0%	0.0%	0.0%	0.0%	0.0%	0.0%	0.0%	0.0%	0.0%	0.0%	0.0%	0.0%	0.0%	0.0%
	k__Bacteria;p__NKB19	0.0%	0.0%	0.0%	0.0%	0.0%	0.0%	0.0%	0.0%	0.0%	0.0%	0.0%	0.0%	0.0%	0.0%	0.0%	0.0%	0.0%	0.0%	0.0%	0.0%	0.0%
	k__Bacteria;p__Nitrospirae	1.1%	1.2%	1.0%	0.7%	0.2%	1.2%	1.4%	1.4%	0.8%	2.4%	1.4%	0.7%	0.7%	1.2%	1.4%	1.1%	0.5%	0.9%	1.4%	1.4%	0.8%
	k__Bacteria;p__OC31	0.0%	0.0%	0.0%	0.0%	0.0%	0.0%	0.0%	0.0%	0.0%	0.0%	0.0%	0.0%	0.0%	0.0%	0.0%	0.0%	0.0%	0.0%	0.0%	0.0%	0.0%
	k__Bacteria;p__OD1	1.3%	1.3%	1.4%	1.4%	0.9%	1.8%	1.2%	0.4%	1.4%	0.3%	0.7%	1.0%	0.5%	1.0%	2.1%	1.1%	1.8%	2.1%	1.1%	2.2%	1.5%
	k__Bacteria;p__OP11	0.1%	0.1%	0.1%	0.1%	0.1%	0.1%	0.1%	0.0%	0.1%	0.0%	0.0%	0.1%	0.1%	0.1%	0.1%	0.1%	0.1%	0.1%	0.1%	0.1%	0.1%
	k__Bacteria;p__OP3	0.1%	0.1%	0.1%	0.1%	0.0%	0.1%	0.1%	0.0%	0.0%	0.0%	0.0%	0.1%	0.0%	0.0%	0.1%	0.1%	0.1%	0.1%	0.1%	0.1%	0.0%
	k__Bacteria;p__Planctomycetes	13.5%	14.3%	15.3%	12.9%	10.1%	14.1%	14.0%	13.7%	13.4%	14.6%	14.6%	12.9%	13.6%	13.2%	13.9%	12.8%	12.1%	13.3%	14.2%	13.7%	13.5%
	k__Bacteria;p__Proteobacteria	24.7%	23.7%	25.9%	24.8%	23.2%	24.3%	25.1%	24.6%	25.6%	24.8%	23.1%	24.5%	23.2%	25.0%	25.1%	23.8%	27.1%	24.6%	26.0%	24.7%	23.9%
	k__Bacteria;p__SR1	0.0%	0.0%	0.0%	0.0%	0.0%	0.0%	0.0%	0.0%	0.0%	0.0%	0.0%	0.0%	0.0%	0.0%	0.0%	0.0%	0.0%	0.0%	0.0%	0.0%	0.0%
	k__Bacteria;p__Spirochaetes	0.0%	0.0%	0.0%	0.0%	0.0%	0.1%	0.0%	0.0%	0.0%	0.0%	0.0%	0.0%	0.0%	0.1%	0.0%	0.0%	0.0%	0.0%	0.0%	0.0%	0.0%
	k__Bacteria;p__TM6	0.1%	0.1%	0.1%	0.1%	0.2%	0.1%	0.1%	0.1%	0.1%	0.1%	0.1%	0.1%	0.2%	0.1%	0.1%	0.1%	0.1%	0.1%	0.1%	0.1%	0.1%
	k__Bacteria;p__TM7	0.8%	0.6%	0.8%	0.9%	0.7%	0.8%	0.7%	0.4%	0.7%	0.4%	0.7%	0.9%	0.8%	0.7%	0.9%	1.1%	1.5%	1.3%	0.9%	0.8%	0.9%
	k__Bacteria;p__Tenericutes	0.0%	0.1%	0.0%	0.0%	0.0%	0.1%	0.0%	0.0%	0.0%	0.0%	0.0%	0.0%	0.0%	0.1%	0.0%	0.2%	0.1%	0.0%	0.1%	0.0%	0.1%
	k__Bacteria;p__Verrucomicrobia	9.1%	9.2%	8.1%	9.5%	11.4%	8.7%	9.1%	10.4%	9.1%	9.4%	9.7%	9.8%	10.0%	8.6%	9.0%	9.5%	7.5%	8.5%	7.9%	7.8%	8.0%
	k__Bacteria;p__WPS-2	0.1%	0.1%	0.1%	0.3%	0.6%	0.0%	0.0%	0.0%	0.3%	0.0%	0.0%	0.2%	0.1%	0.0%	0.0%	0.2%	0.3%	0.2%	0.0%	0.1%	0.2%
	k__Bacteria;p__WS2	0.0%	0.0%	0.0%	0.0%	0.0%	0.0%	0.0%	0.0%	0.0%	0.0%	0.0%	0.0%	0.0%	0.0%	0.1%	0.0%	0.0%	0.1%	0.0%	0.0%	0.0%
	k__Bacteria;p__WS3	0.4%	0.4%	0.4%	0.2%	0.0%	0.8%	0.7%	0.4%	0.6%	0.8%	0.5%	0.1%	0.1%	0.6%	0.5%	0.5%	0.2%	0.2%	0.5%	0.6%	0.4%
	k__Bacteria;p__WS4	0.0%	0.0%	0.0%	0.0%	0.0%	0.0%	0.0%	0.0%	0.0%	0.0%	0.0%	0.0%	0.0%	0.0%	0.0%	0.0%	0.0%	0.0%	0.0%	0.0%	0.0%
	k__Bacteria;p__WS5	0.0%	0.0%	0.0%	0.0%	0.0%	0.0%	0.0%	0.0%	0.0%	0.0%	0.0%	0.0%	0.0%	0.0%	0.0%	0.0%	0.0%	0.0%	0.0%	0.0%	0.0%
	k__Bacteria;p__WS6	0.0%	0.0%	0.0%	0.0%	0.0%	0.0%	0.0%	0.0%	0.0%	0.0%	0.0%	0.0%	0.0%	0.0%	0.0%	0.0%	0.0%	0.0%	0.0%	0.0%	0.0%
	k__Bacteria;p__[Caldithrix]	0.0%	0.0%	0.0%	0.0%	0.0%	0.0%	0.0%	0.0%	0.0%	0.0%	0.0%	0.0%	0.0%	0.0%	0.0%	0.0%	0.0%	0.0%	0.0%	0.0%	0.0%

Figure S3 Counts of P genes by PICRUSt and GeoChip scaled independently.

Black = PICRUSt. Red = Geochip. Separated for scale.

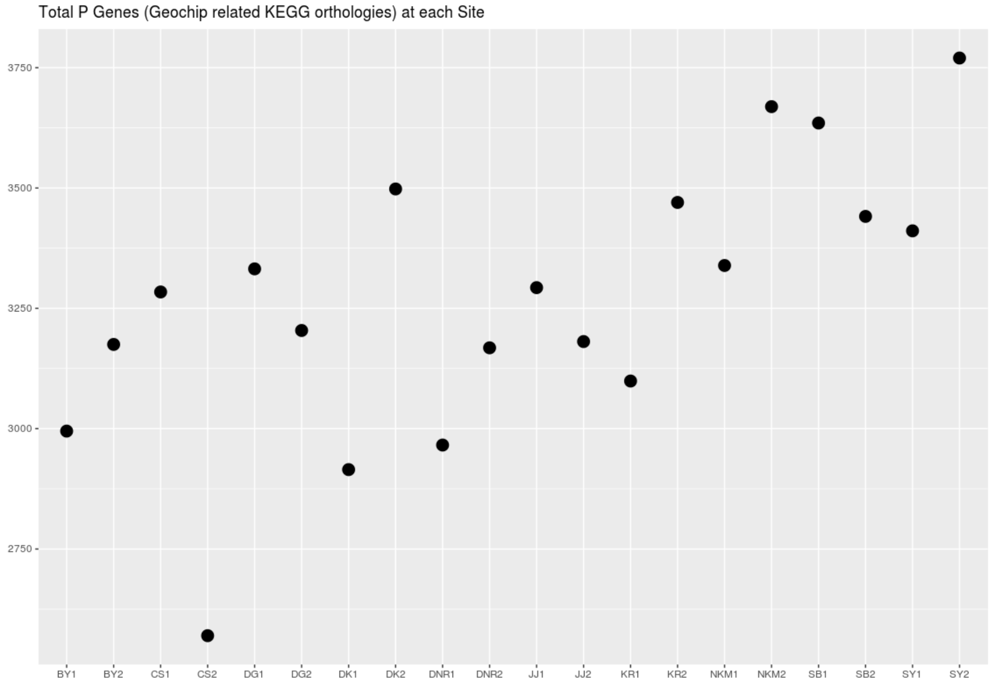

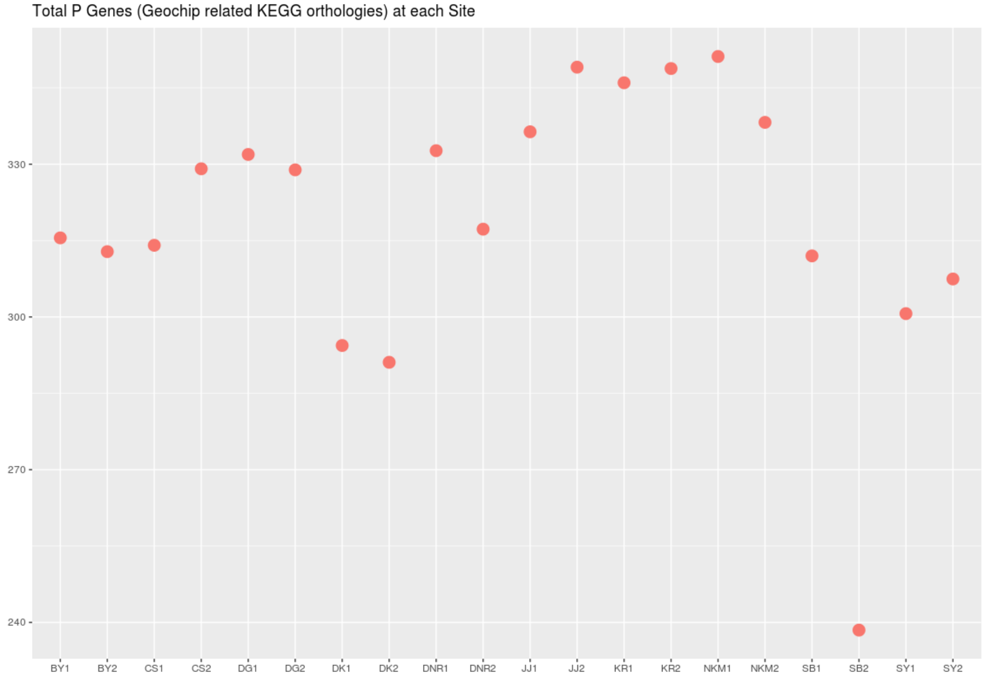

Figure S4 Procrustes Error Plot for Procrustes analysis between PICRUSt and GeoChip datasets.

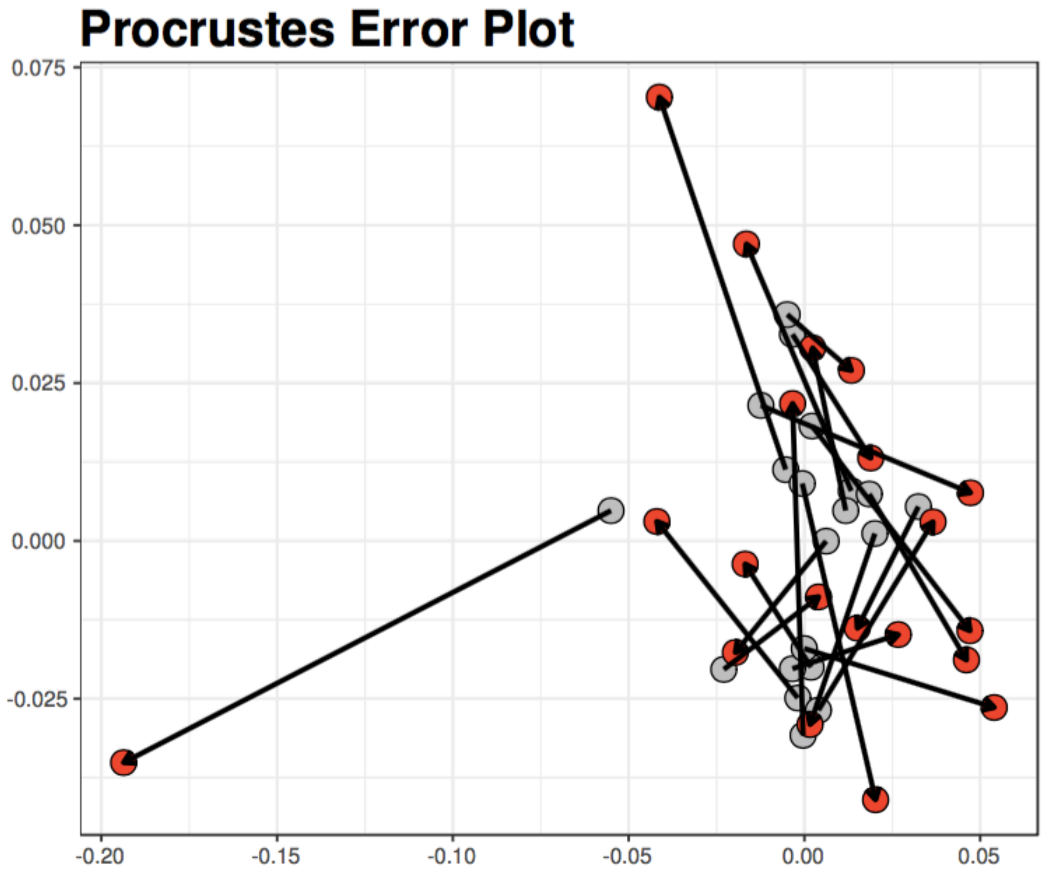

Figure 5 A) Bubble plot of taxa identified by new assay at each site. B) Total counts identified in new assay at each site by PICRUSt.

A)

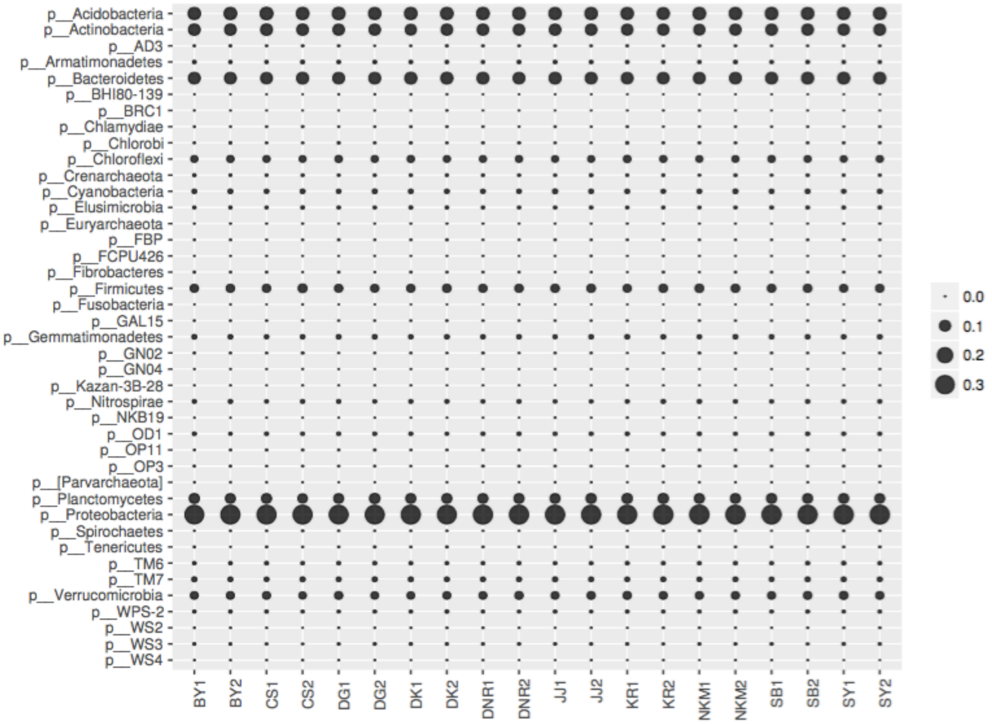

B)

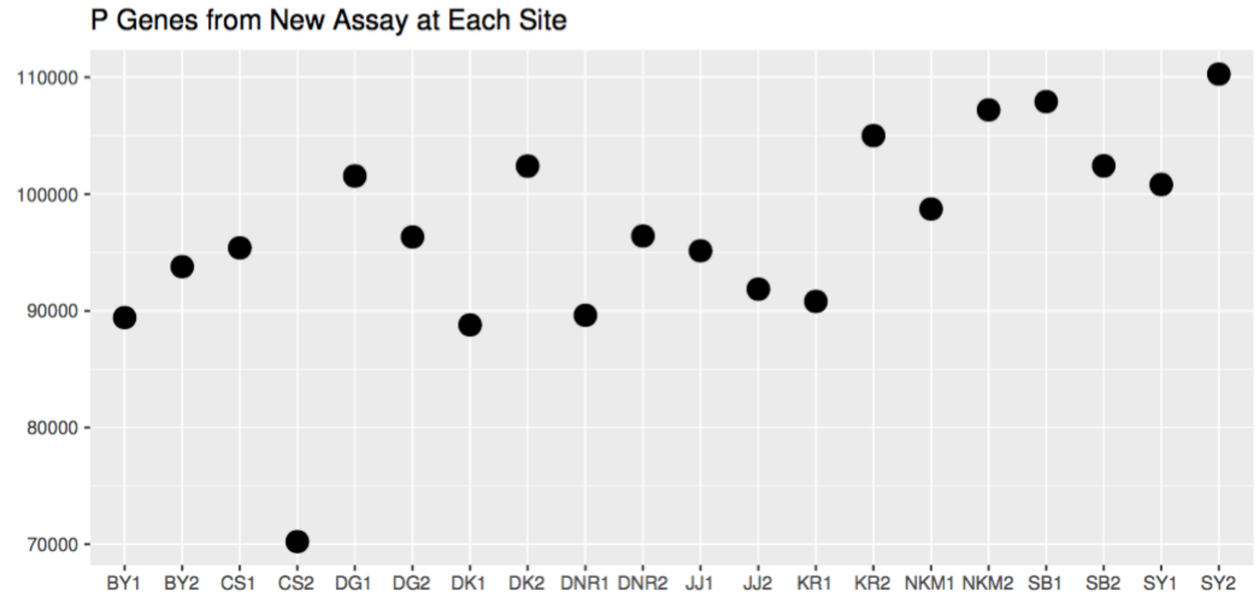

Supplement: Supplementary file 1 [file f1000research-7-15044-s0000.tgz › 336db551-5863-498c-85f8-773bc96fbbe8.pdf]
