# Supplementary material for: A comparison of computationally predicted functional metagenomes and microarray analysis for microbial P cycle genes in a unique basalt-soil forest
Source: F1000Res. 2018 Feb 12;7:179. [Version 1] doi: 10.12688/f1000research.13841.1 (PMC6051228; doi:10.12688/f1000research.13841.1)
Supplement: Supplementary file 2 [file f1000research-7-15044-s0001.tgz › 489a9fce-c24d-4f28-a684-fc8ea7feef41.pdf]

Supplementary Information: Supplementary Table

A comparison of computationally predicted functional metagenomes and microarray analysis for microbial P cycle genes in a unique basalt-soil forest.

Erick S. LeBrun[1]* and Sanghoon Kang[1]

[1] Center for Reservoir and Aquatic Systems Research, Department of Biology, Baylor University, Waco, Texas 76798-7388 USA

*Corresponding author:

Erick S. LeBrun

Department of Biology

Baylor University

One Bear Place 97388

Waco, Texas 76798-7388 USA

Erick_LeBrun@Baylor.edu

Conflict of Interest Statement:

The authors declare no conflict of interest.

Supplementary Table

Table S1 KEGG Orthologies included in “new” PICRUSt P assay.

Gene/Enzyme	KEGG Orthology Numbers						
phnA	K06193	K19670					
phnM	K06162						
phnB	K04750						
phnH	K06165						
phnI	K06164						
phnO	K09994						
phnJ	K06163						
phnK	K05781						
RcsF	K06080						
phnF	K02043						
phoP	K07660	K07658					
pstC	K02037						
pstA	K02038						
phoR	K07636						
phoB	K07657	K07657					
pstB	K02036						
phoU	K05946						
ppk	K00937						
plsC	K00655						
phoA	K01077						
lnt	K03820						
ppX1	K01514						
ppaX	K06019						
ppa	K01507						
yggT	K02221						
pit	K03306						
corC	K06189						
ppaC	K15986						
ybeY	K07042						
pntA	K00324						
pntB	K00325						
nnt	K00323						
Phosphatase	K00906	K01514	K05307	K06366	K08743	K13988	K16054
	K01077	K01515	K05518	K06367	K08966	K14019	K16055
	K01078	K01517	K05602	K06368	K09474	K14165	K16340

	K01079	K01518	K05693	K06369	K09612	K14379	K16662
	K01080	K01519	K05694	K06478	K09880	K14394	K16667
	K01084	K01520	K05695	K06645	K10047	K14395	K16723
	K01085	K01521	K05696	K06776	K10147	K14410	K16833
	K01086	K01522	K05697	K06777	K10909	K14497	K16904
	K01087	K01524	K05698	K06778	K10916	K14501	K16910
	K01089	K01525	K05766	K06881	K11240	K14634	K17453
	K01090	K01526	K05866	K06896	K11532	K14803	K17457
	K01091	K01622	K05867	K06928	K11583	K14819	K17458
	K01092	K02226	K05978	K06949	K11584	K15422	K17459
	K01093	K02374	K05979	K07024	K11725	K15423	K17491
	K01094	K02446	K06018	K07026	K11751	K15424	K17499
	K01095	K02555	K06019	K07053	K11777	K15425	K17500
	K01096	K03084	K06116	K07189	K11915	K15426	K17501
	K01097	K03103	K06117	K07252	K11938	K15427	K17502
	K01098	K03270	K06124	K07293	K12152	K15494	K17503
	K01099	K03273	K06153	K07313	K12328	K15498	K17504
	K01100	K03426	K06162	K07314	K12329	K15499	K17505
	K01101	K03456	K06268	K07315	K12354	K15500	K17506
	K01102	K03574	K06269	K07658	K12584	K15501	K17507
	K01103	K03788	K06270	K07757	K12804	K15502	K17508
	K01104	K03841	K06352	K07758	K12944	K15503	K17509
	K01106	K04041	K06353	K07766	K12945	K15504	K17549
	K01107	K04342	K06354	K07817	K12977	K15529	K17550
	K01109	K04348	K06355	K08050	K12978	K15544	K17552
	K01110	K04354	K06356	K08067	K13084	K15637	K17553
	K01111	K04382	K06357	K08073	K13085	K15640	K17555
	K01112	K04457	K06358	K08075	K13086	K15728	K17556
	K01113	K04458	K06359	K08077	K13216	K15731	K17557
	K01122	K04459	K06360	K08114	K13248	K15732	K17558
	K01139	K04460	K06361	K08296	K13297	K15759	K17562
	K01507	K04461	K06362	K08310	K13555	K15781	K17565
	K01509	K04486	K06363	K08312	K13617	K15850	K17566
	K01512	K04716	K06364	K08320	K13807	K15909	K17567
	K01513	K04765	K06365	K08726	K13987	K15986	K17568
phoH	K06217						
oprO_P	K07221						
hppA	K15987						
phoQ	K07637						
SLC20A	K14640						

sthA	K00322
phnW	K03430
EC4.1.1.82	K09459
phnX	K05306
