# Supplementary material for: A comparison of computationally predicted functional metagenomes and microarray analysis for microbial P cycle genes in a unique basalt-soil forest
Source: F1000Res. 2018 Feb 12;7:179. [Version 1] doi: 10.12688/f1000research.13841.1 (PMC6051228; doi:10.12688/f1000research.13841.1)
Supplement: Peer review history

# Peer review: Referee response for version 1

**DOI:** 10.5256/f1000research.15044.r32731

**Published:** 2018-07-17

**Authors:** Joy D Van Nostrand

**Affiliations:** 1 University of Oklahoma, Norman, Oklahoma, USA

LeBrun and Kang compared two metagenomic functional gene datasets of the same microbial community, functional gene microarray and PICRUSt 16S rRNA sequencing data.  PICRUSt detected a much higher gene diversity and abundance than the microarray.  Some of that difference could be accounted for by group probes on the GeoChip microarray that cover two or more similar sequences, although PICRUSt offers a much larger database.  The authors make a good point about the relative ease of expanding coverage by including additional genes for the PICRUSt method.  Microarray require additional steps and manufacturing a new array compared to simply updating a database for PICRUSt.  The methods used and the conclusions are sound.  This paper can be indexed.

 Minor edit - The second Kim et al. reference in the methods section has an incorrect citation - I believe it should be 10 (or 15)

I have read this submission. I believe that I have an appropriate level of expertise to confirm that it is of an acceptable scientific standard.

# Peer review: Referee response for version 1

**DOI:** 10.5256/f1000research.15044.r30837

**Published:** 2018-03-23

**Authors:** Ye Deng

**Affiliations:** 1 CAS Key Laboratory of Environmental Biotechnology, Research Center for Eco-Environmental Sciences, Chinese Academy of Sciences, Beijing, China

The authors reported a parallel comparison on two most popular high-throughput technologies on environmental microbiota studies, 16S sequencing and GeoChip. Other than taxa aspect, they focused on the comparison of functional gene involving in P. Overall, the methods and results are sound that 16S sequencing detected more taxa involving in P since 417 KO could be predicted by PtCRUST rather than 3 KO in GeoChip. It is unsurprising because functional gene microarray can only pick up limited number of genes in each bio-process as marked functional traits, while the high-throughput sequencing can be much more open to uncultured and novel taxa in environments. Zhou *et al.* already mentioned this opinion in their review paper ^1^ as well. Thus, according to the policy of F1000, I think this paper can be indexed.

I have read this submission. I believe that I have an appropriate level of expertise to confirm that it is of an acceptable scientific standard.

## References

[1] ZhouJHeZYangYDengYTringeSGAlvarez-CohenL: High-throughput metagenomic technologies for complex microbial community analysis: open and closed formats. *MBio*.2015;6(1) : 10.1128/mBio.02288-14 10.1128/mBio.02288-14 25626903PMC4324309
